# Current opinions on autophagy in pathogenicity of fungi

**DOI:** 10.1080/21505594.2018.1551011

**Published:** 2018-12-03

**Authors:** Xue-Ming Zhu, Lin Li, Min Wu, Shuang Liang, Huan-Bin Shi, Xiao-Hong Liu, Fu-Cheng Lin

**Affiliations:** State Key Laboratory for Rice Biology, Institute of Biotechnology, Zhejiang University, Hangzhou, China

**Keywords:** Autophagy, endocytosis, signaling pathway, crosstalk, interaction

## Abstract

The interaction between pathogens and their host plants is a ubiquitous process. Some plant fungal pathogens can form a specific infection structure, such as an appressorium, which is formed by the accumulation of a large amount of glycerin and thereby the creation of an extremely high intracellular turgor pressure, which allows the penetration peg of the appressorium to puncture the leaf cuticle of the host. Previous studies have shown that autophagy energizes the accumulation of pressure by appressoria, which induces its pathogenesis. Similar to other eukaryotic organisms, autophagy processes are highly conserved pathways that play important roles in filamentous fungal pathogenicity. This review aims to demonstrate how the autophagy process affects the pathogenicity of plant pathogens.

## Introduction

Autophagy is an evolutionarily conserved process in eukaryotes. To maintain or restore homeostasis under stress and thus aid plant survival, some of the proteins or organelles that are damaged during the autophagy process are engulfed by autophagic vesicles with double-membrane structures and shuttled to lysosomes (animals) or vacuoles (fungi and plants) for degradation and recycling [1,2]. Since the first ATG gene, ATG1, termed the first autophagy-related gene, was found in 1992, approximately 36 ATG proteins that drive the autophagic process have been identified in *Saccharomyces cerevisiae* [3]. Among these ATG genes, *ATG1-9, ATG12-14, ATG16*, and *ATG18* are essential for the formation of autophagic vesicles [4,5]. According to their specific functions, these Atg proteins have been classified into four major groups: the Atg1 kinase complex, the ubiquitin-like Atg8/Atg12 conjugation systems, the phosphatidylinositol 3-phosphate kinase complex, and the Atg9 recycling complex [6–10].

In recent decades, many studies have indicated that autophagy plays an important role in the pathogenicity of plant pathogens. An improved understanding of the molecular mechanisms underlying plant fungal pathogenesis and their interface with autophagic processes will ultimately lead to an improved management of plant fungal diseases. Here, we review the current knowledge on autophagy and discuss recent findings regarding plant pathogenic fungi and the functional links between autophagy and fungal pathogenesis in plants.

## Interactions between pathogenic fungi and plants

**Figure 1.**
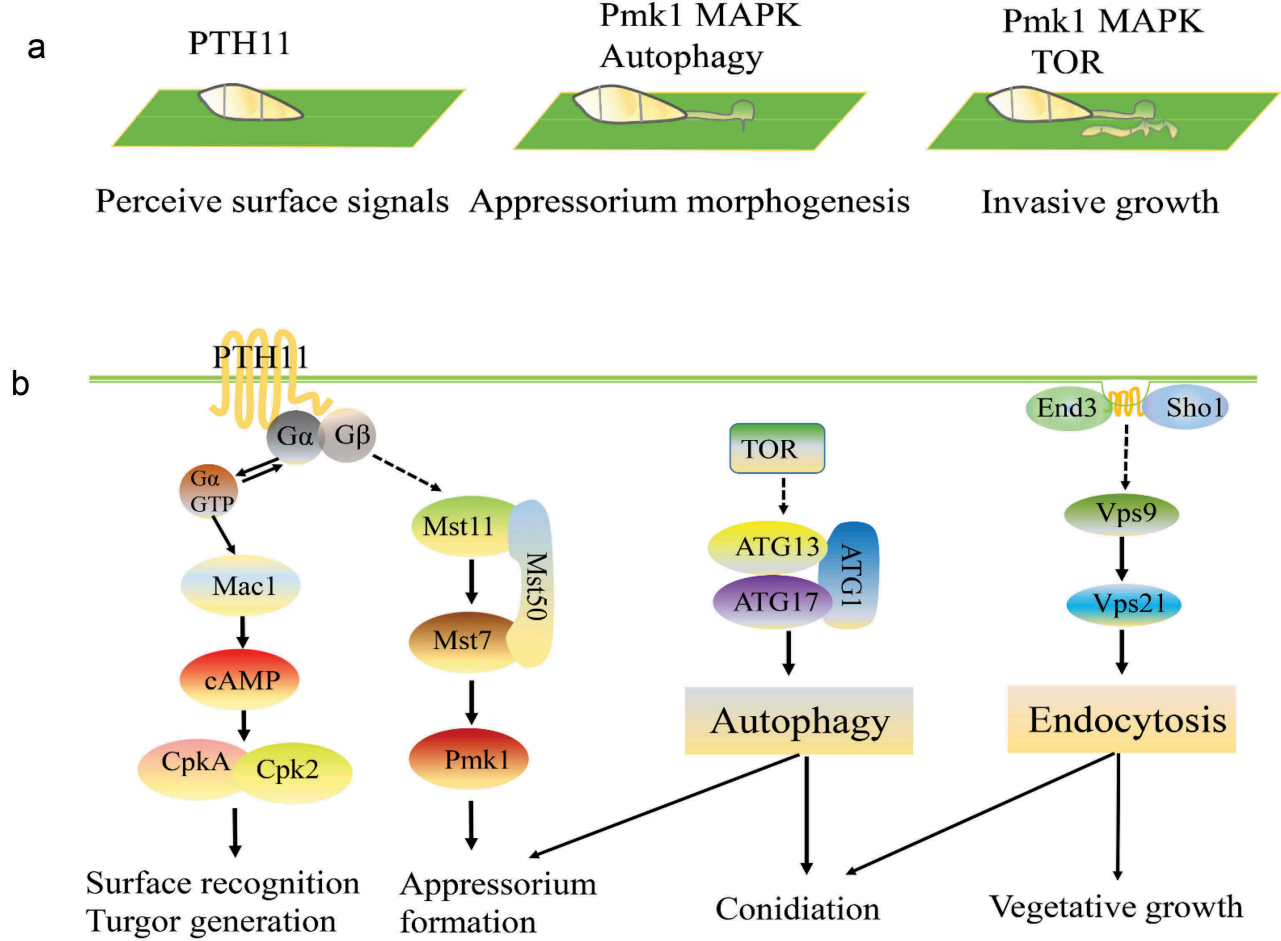
Several signaling pathways involved in appressorium formation and pathogenicity in *Magnaporthe oryzae.* a. The Pmk1 MAPK and autophagy pathways are involved in different stages of the plant infection process. b. Summary of the detection and transmission of fungal pathogenesis-related proteins to the outside signals. Pth11 can recognize surface signals, such as surface hydrophobicity and hardness, and activate downstream Mst11-Mst7-Pmk1 MAPK and endosomal pathways. These two pathways regulate appressorium formation, penetration, and invasive growth. TOR (Target of rapamycin) recognizes extracellular signals, such as nitrogen, glucose and some environmental stresses, and regulates autophagy by controlling the Atg1-Atg13-Atg17 complex.

In recent years, the mechanisms underlying the interactions between plants and fungi have been a hot topic in plant pathology research, and the corresponding results should broaden the theoretical foundation of plant resistance mechanisms and aid the breeding of resistant plants. Diverse plants generate different obstacles to block infection by potential fungal pathogens, such as physical structures and molecular substances [11]. Pathogen infection often results in the induction of defense signaling in the local infected tissue and the formation of different infection-related structures that can break through the host plant’s roots, stems, leaves, flowers or other special tissues [2,11,12]. In the past decade, *Magnaporthe oryzae* and *Colletotrichum* spp. have been used as models for studying the interactions between plants and pathogenic fungi. The most important infection structures in *M. oryzae* and *Colletotrichum* spp. are the appressoria, which are required for infection. In the context of host–pathogen interactions, the cell surface represents the primary site of interaction between the two organisms and is the location where the majority of infection-related events, such as mutual recognition, pathogen adhesion and penetration, and the degradation of host substrates, take place. Several signaling pathways are involved in appressorium formation and pathogenicity in *M. oryzae* (Figure 1(a,b)). The PKA mitogen-activated protein kinase (MAPK) pathway participates in the identification of the host surfaces, and the Pmk1 MAPK pathway is essential for appressorium formation and invasive growth [13].

The latest research shows that the TOR pathway is also involved in the cell cycle and autophagic cell death. Marroquin-Guzman et al. showed that glucose-ABL1-TOR signaling modulates the cell cycle to control terminal appressorial cell differentiation [14]. Kim et al. showed that TOR regulates some effectors for cellular survival in *Aspergillus nidulans* undergoing rapamycin-induced and carbon starvation-induced autophagy [15]. Furthermore, an increasing amount of research indicates that the autophagy and endocytosis pathways are also essential for conidial and appressorium formation [16–19].

## Biological process and function of autophagy

**Figure 2.**
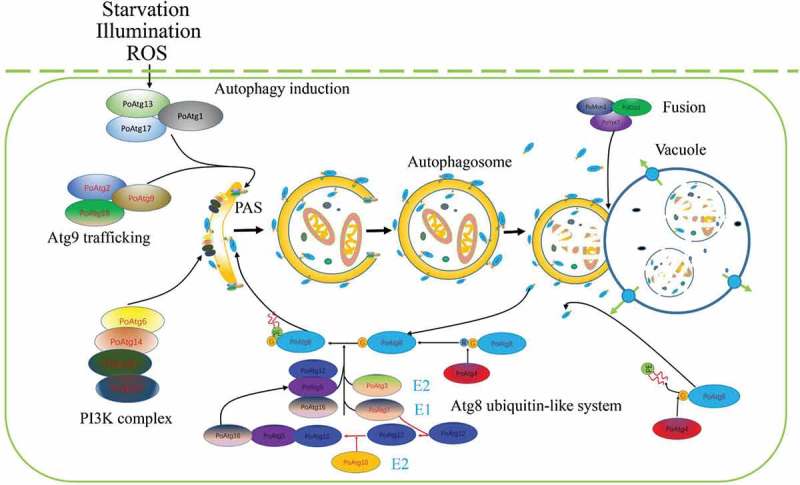
Autophagy process in filamentous fungi. After the induction of autophagy, an initial sequestering phagophore is assembled at the phagophore assembly site (PAS). Expansion and curvature of the phagophore leads to engulfment of the cargo (cytoplasm containing proteins and organelles) into the double-membraned autophagosome. The fusion of the autophagosomal outer membrane with the vacuolar membrane results in the release of the autophagic body, which is surrounded by the inner autophagosomal membrane. Autophagic bodies and their cargoes are degraded by hydrolytic enzymes, and the degraded products are exported into the cytoplasm for reuse.

Similar to yeast and mammals, autophagy occurs in pathogenic fungi and mediates the incorporation of the cytoplasm and organelles into lysosomes/vacuoles for degradation, which allows the targeting of cytoplasm and organelles as nutrients to support cell survival. Evidence obtained over the last 10 years shows that autophagy is not only important for filamentous growth under starvation and non-starvation conditions but also often a prerequisite for pathogenicity (Table 1). For example, the autophagy pathways in *M. oryzae* are primarily composed of the following five systems: an Atg1-Atg13-Atg17 complex, an Atg9 trafficking system, a PI3K complex and two ubiquitin-like systems (Figure 2). If rice blast fungus is exposed to external environmental stress (e.g., lack of nutrients, injury, lack of oxygen, and ROS accumulation) or is treated with rapamycin, the activity of TOR, a regulatory protein upstream of autophagy, is decreased, resulting in the dephosphorylation of Atg13, the subsequent binding of Atg17 and the activation of Atg1. Atg1 forms the Atg1-Atg13-Atg17 complex and is localized at the autophagy initiation site (phagophore assembly site (PAS)), which marks the initiation of autophagy [20]. Although the Atg1-Atg13-Atg17 complex is essential for the formation of autophagosomes in yeast or mammals, it has not been found to have any influence on the pathogenicity of *M. oryzae*, as demonstrated by the lack of effects observed after the knockout of *MoATG13* or *MoATG17* but not *MoATG1* [21]. After the induction of autophagy, the autophagy membrane extends to form vesicles at the PAS, which eventually results in the formation of autophagosomes, and many proteins and membrane materials participate in the extension, fusion and expansion of autophagosomes. Among the membrane components participating proteins, Atg9 is believed to be the main factor of the source of the autophagic membrane, but the formation of autophagic vesicles also requires the participation of other proteins. Specifically, Atg2, Atg18 and Atg9 interact, which affects the positioning of Atg9 [22]. Recent studies have shown that Atg9 proteins can assemble into a capsule-like vesicular structure composed of approximately 30 Atg9 molecules, and this structure induces formation of the autophagosome by providing the main source of the membrane. After formation of the autophagosome, the Atg9 molecules automatically move off the autophagy body to undergo repeated recycling and avoid being transported into vacuoles for degradation [23,24].

**Table 1.** Core autophagy genes related to pathogenicity in filamentous fungi.

Gene	Species	Conidiation	Pathogenicity	References
ATG1-ATG13-ATG17 complex				
MoATG1	*Magnaporthe oryzae*	Reduced	Lost	[20]
BbATG1	*Beauveria bassiana*	Reduced	Reduced	[25]
BcATG1	*Botrytis cinerea*	Reduced	Lost	[26]
FgATG1	*Fusarium graminearum*	Reduced	Reduced	[27]
MoATG13	*Magnaporthe oryzae*	Normal	Normal	[45]
AoATG13	*Aspergillus oryzae*	Slightly reduced	Not mentioned	[28]
FgATG3	*Fusarium graminearum*	Reduced	Reduced	[27]
MoATG17	*Magnaporthe oryzae*	Reduced	Lost	[21]
FgATG17	*Fusarium graminearum*	Normal	Normal	[27]
AnATG17	*Aspergillus niger*	Normal	Not mentioned	[29]
PI3K complex				
MoATG6	*Magnaporthe oryzae*	Lost	Lost	[16]
PsATG6a	*Phytophthora sojae*	Reduced	Reduced	[30]
FgATG6	*Fusarium graminearum*	Reduced	Reduced	[27]
PsATG6b	*Phytophthora sojae*	Normal	Normal	[30]
MoATG14	*Magnaporthe oryzae*	Reduced	Lost	[35]
FgATG14	*Fusarium graminearum*	Reduced	Reduced	[27]
SmVPS15	*Sordaria macrospora*	Reduced germination rates	Not mentioned	[31]
SmVPS34	*Sordaria macrospora*	Reduced germination rates	Not mentioned	[31]
ATG9 trafficking				
MoATG2	*Magnaporthe oryzae*	Reduced	Lost	[32]
FgATG2	*Fusarium graminearum*	Reduced	Reduced	[27]
MoATG9	*Magnaporthe oryzae*	Reduced	Lost	[45]
FgATG9	*Fusarium graminearum*	Reduced	Decrease Reduced	[27,64]
MoATG18	*Magnaporthe oryzae*	Reduced	Lost	[16]
FgATG18	*Fusarium graminearum*	Reduced	Reduced	[27]
Ubiquitin-like system				
MoATG3	*Magnaporthe oryzae*	Reduced	Lost	[21]
FgATG3	*Fusarium graminearum*	Reduced	Reduced	[27]
BcATG3	*Botrytis cinerea*	Reduced	Lost	[33]
MoATG7	*Magnaporthe oryzae*	Reduced	Lost	[21]
BcATG7	*Botrytis cinerea*	Reduced	Lost	[33]
FgATG7	*Fusarium graminearum*	Reduced	Reduced	[27]
MoATG10	*Magnaporthe oryzae*	Reduced	Lost	[21]
MoATG5	*Magnaporthe oryzae*	Reduced	Lost	[21]
FgATG10	*Fusarium graminearum*	Reduced	Reduced	[27]
MoATG12	*Magnaporthe oryzae*	Reduced	Lost	[21]
SmATG12	*Sordaria macrospora*	Impaired growth	Not mentioned	[34]
FgATG12	*Fusarium graminearum*	Reduced	Reduced	[27]
MoATG16	*Magnaporthe oryzae*	Reduced	Lost	[16]
FgATG16	*Fusarium graminearum*	Reduced	Reduced	[27]
MoATG4	*Magnaporthe oryzae*	Reduced	Lost	[21]
FgATG4	*Fusarium graminearum*	Reduced	Reduced	[27]
MoATG8	*Magnaporthe oryzae*	Reduced	Lost	[21]
FgATG8	*Fusarium graminearum*	Reduced	Reduced	[27]

In *M. oryzae*, the PI3K kinase complexes are divided into two categories. The first category is comprised of Atg6, Atg14, Vps34 and Vps15, which induce autophagosome formation, and the extension and expansion of phagosomes results in the recruitment of many downstream proteins, such as Atg2, Atg18, Atg20, and Atg24 [35]. The second category consists of Atg6, Vps38, Vps34 and Vps15, which are mainly involved in the endocytic pathway. It was recently reported that in nematodes, Atg6 regulates the processes of endocytosis and autophagy through the competitive binding of Vps38 and Atg14, and crosstalk exists between these two pathways, i.e., the autophagy pathway is promoted if the endocytic pathway is inhibited. This phenomenon has also been observed in fungi [19].

Two types of ubiquitin-like systems, the Atg8-PE ubiquitin system and the Atg12-Atg5-Atg16 ubiquitin-like system, play crucial roles in the extension and expansion of autophagosomes. In the rice blast fungus, the Atg8 protein consists of 121 amino acid residues (a glycine is found at residue 116); under normal conditions, Atg8 is cleaved by Atg4 to yield a 116-amino-acid-residue protein [36]. The Atg7 and Atg3 proteins act on Atg8, similar to E1 (ubiquitin-activating) and E2 (ubiquitin-conjugating) enzymes in the ubiquitination pathway, respectively [22]. The second ubiquitin-like protein Atg12 that linked to an internal lysine of Atg5 by C-terminal glycine and then Atg12-Atg5 conjugate binds Atg16 to form Atg12-Atg5-Atg16 complex. The Atg12-Atg5-Atg16 complex functions as an E3 ubiquitin ligase that ultimately conjugates Atg8 with PE (phosphatidyl ethanolamine) molecules; this conjugation allows the molecules to be anchored on the outer membrane of autophagic vesicles and thereby aid the extension and expansion of autophagosomes [8].The combined action of a variety of protein molecules and membrane materials eventually results in the formation of autophagosome vesicles, and mature autophagosomes need to fuse with vacuoles to complete their mission. During the fusion process, many proteins, such as SNARE (soluble N-ethylmale-imide-sensitive factor-attachment protein receptors) and Rab-related proteins, are also required, and the Atg8-PE molecules on the outer autophagic membrane are removed from the membrane by Atg4, which allows the recycling of Atg8 and the vacuolar degradation of the inner membrane and its enclosed substances [8,35,37].

Many other Atg proteins in pathogenic fungi are involved in selective autophagy, such as mitophagy (selective degradation of mitochondria via autophagy and it is an important mitochondrial quality control mechanism), pexophagy (degradation of peroxisomes and it participates in peroxisomal quality control both under normal growth conditions and during starvation), and cytoplasm-to-vacuole targeting (CVT) pathway. Atg11, Atg13, Atg101, and Atg24 reportedly participate in mitophagy in *M. oryzae* and *A. oryzae* [16,21,38,39]; Atg26, Atg28, and Atg36 mediate pexophagy in *C. orbiculare* [40]; and Atg19, Atg21, Atg23, Atg27, and Atg34 participate in the CVT pathway [21]. Functional studies of these autophagy-related (ATG) genes performed over the past decade (Table 1) have revealed an important link between autophagy and fungal development. Recent studies have also shown that some non-ATG genes are involved in the autophagy pathway (Table 2). In mammals, Rab1, Rab5, and Rab11 play important roles in the regulation of autophagy by regulating mTOR activity; specifically, these three proteins are involved in autophagosome formation and autophagosome-lysosome fusion [41]. Recent studies have also demonstrated that some endocytosis-related genes, including MoYpt7, PoVps35, PoVps26, PoVps17, MoVps41, and MoEnd3, are involved in autophagy in *M. oryzae* [17,37,42,53].

**Table 2.** New factors that regulate autophagy in filamentous fungi.

Gene	Species	Phenotype	Functions	References
TOR	*Magnaporthe oryzae*	Impaired growth	Supports cell cycle progression and inhibits autophagy	[60]
MoSNT2	*Magnaporthe oryzae*	Impaired growth and conidiation	Regulates infection-associated autophagy	[60]
MoVPS35	*Magnaporthe oryzae*	Impaired generation of appressorium turgor	Involved in conidial autophagic cell death	[42]
MoVPS17	*Magnaporthe oryzae*	Impaired growth and conidiation	Involved in conidiation and conidial morphology	[17]
MoYPT7	*Magnaporthe oryzae*	Impaired growth and conidiation	Required for membrane fusion in autophagy	[37]
FgRAB7	*Fusarium graminearum*	Impaired vegetative growth	Essential for autophagy-dependent development	[65]
MoRAB5	*Magnaporthe oryzae*	Impaired growth and conidiation	Involved in autophagic closure	[54]
FgMON1	*Fusarium graminearum*	Impaired growth and conidiation	Involved in autophagic fusion	[52]
MoGCN5	*Magnaporthe oryzae*	Production of abundant conidia, but these conidia are unable to infect the host successfully	Negatively regulates light- and nitrogen starvation-induced autophagy	[43]
MoEND3	*Magnaporthe oryzae*	Impaired appressorium formation and virulence	Involved in autophagic degradation	[53]
MoDNM1	*Magnaporthe oryzae*	Impaired appressorium formation and virulence	Involved in mitophagy	[44]

## Autophagy and endocytosis in pathogenesis

**Figure 3.**
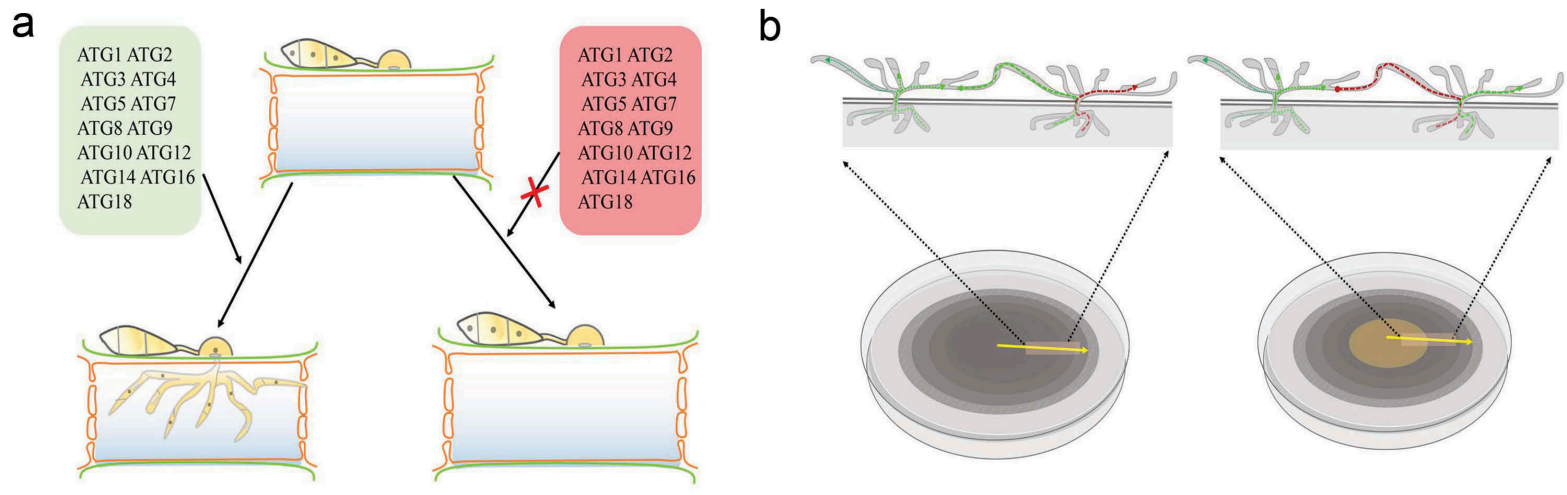
Autophagy mediates the infection of filamentous fungi and cell survival. a. The deletion of any of the core ATG genes in filamentous fungi results in defects in fungal penetration into the host. The autophagy-deficiency mutants lose their ability to cause disease in their host plants. b. In plate culture, the cells of autophagy-deficiency mutants lose their ability to recycle the materials produced by the cells. As a result, nutrients can no longer be transmitted between hyphal cells, and the middle sections of old hyphae collapse due to nutrient depletion.

The mechanism through which the autophagy pathways affect the pathogenicity of pathogenic fungi is a topic that has been investigated by many researchers in recent years. In *M. oryzae*, the knockout of any of the core Atg genes (MoATG1-12, MoATG14-16, MoATG18), with the exception of *MoATG13* and *MoATG17*, results in the loss of pathogenicity, and this finding highlights a notable difference between rice blast fungus and yeast [45]. Conidiation and appressorium formation are essential for the host invasion of most filamentous fungi, and the blockage of autophagy significantly reduces the conidiation of *M. oryzae*. Although appressoria can be formed by conidia of *M. oryzae* mutants, these structures cannot penetrate the epidermis of the host plants to cause disease [2,20]. On the one hand, the absence of an intact autophagy machinery affects the expression of transcription factors that regulate conidiation [46–48]; on the other hand, appressoria are formed in autophagy mutants but are unable to penetrate the plant leaves. In *M. oryzae*, appressorium turgor is obtained as a consequence of the accumulation of an enormous quantity of glycerol in a central vacuole of the cell, and this effect generates extremely high pressure (reaching 8 MPa) and thereby allow the penetration peg to penetrate the epidermis of the host [49]. However, the formation of appressoria filled with glycerol has not been observed in *MoATG2, MoATG4, MoATG5, MoATG9* or *MoATG18* gene-deletion mutants of *M. oryzae*, and thus, these mutants are unable to generate sufficient pressure to allow their penetration into the plant leaves. Thus, it has been confirmed that autophagy is essential for the generation of appressorium turgor in *M. oryzae*, and this phenomenon is one of the main reasons explaining why autophagy pathways affect the pathogenicity of fungi (Figure 3(a)). Unlike yeast, filamentous fungi are multicellular organisms, and the growth of new hyphae depends on the nutrients transported by the old hyphae. If the autophagy pathway is blocked, the inability of the aging hyphal organs to be degraded results in the accumulation of reactive oxygen species, and an insufficient use of nutrients leads to hyphal collapse [38,50,51] (Figure 3(b)). Recent studies have shown that the endocytosis pathway also plays an important role in pathogenicity. In *M. oryzae*, the endocytosis-related proteins MoYpt7, PoRab5, PoVps9, MoEnd3, and MoMon1 affect vegetative growth, conidiation, appressorium formation and virulence [19,52–54]. In terms of pathogenic mechanisms, PoVps9 acts as a guanylate exchange factor with Rab5 to regulate the activity of PoRab5 and participate in the formation of the early endosome. The loss of either Vps9 or Rab5 causes a significant decrease in endosome formation in *M. oryzae* [19]. Liu et al. showed that MoYpt7 is essential for the fusion of the late endosome with the vacuole [37], and recent studies have revealed that the endocytosis regulation factor MoEnd3 mediates endocytic transport and is critical for the signal transduction-regulated development and virulence of *M. oryzae* []. These defects affect pathogenic development.

## Crosstalk between autophagy and endocytosis in pathogenic fungi

**Figure 4.**
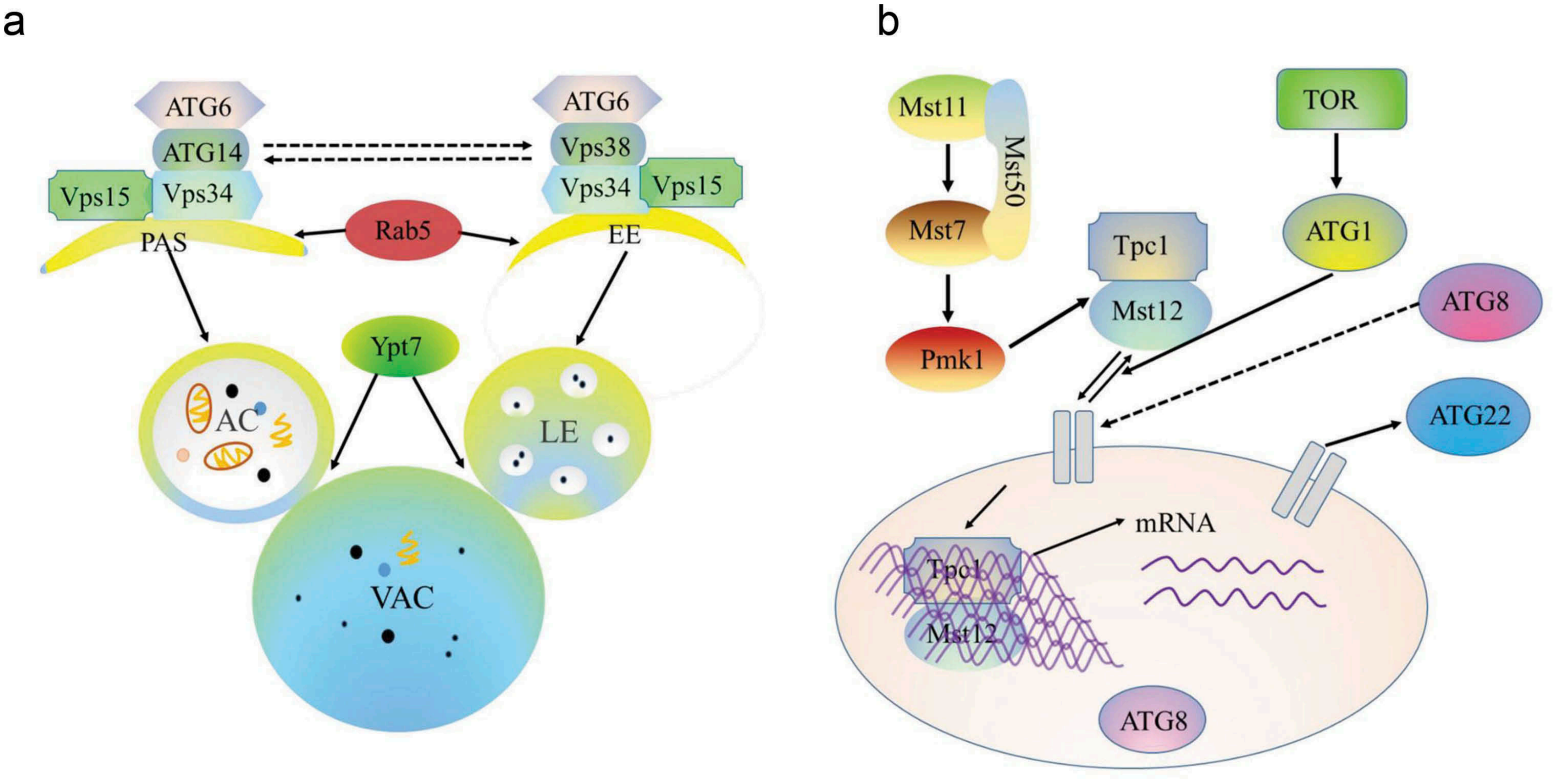
Crosstalk among autophagy and endocytosis and the Pmk1 MAPK pathway. a. Vps34 recruits Atg6 under the action of Vps38 to induce endosomal endocytosis. Simultaneously, Atg6 is recruited by Vps34 under the action of Atg14 to target the PAS, which participates in the autophagy pathway. Both autophagosome and endosome fusion with the vacuole require the Ypt7 protein. PAS, phagophore assembly site; EE, early endosome; AC, autoplastic vacuole; LE, late endosome; VAC, vacuole. b. In *M. oryzae*, environmental signals trigger the Pmk1 kinase cascade to control autophagy and activate Tpc1 function. Nuclear-localized Tpc1 then activates the transcription of genes required for polar growth, autophagy, and glycogen degradation.

In *M. oryzae*, hyphae growth, sporulation and infectivity are regulated by many factors. Many studies have researched several pathogenic pathways in rice blast fungi, including cAMP signaling, which is associated with surface recognition and appressorium formation, and the Pmk1 pathway, which is essential for appressorium formation and plant invasion [55]. Recent research shows that the endocytosis pathway is required and plays a major role in rice blast fungal morphogenesis, vacuole fusion, stress resistance and pathogenicity [17,19,35]. However, until recently, few studies have elaborated on the crosstalk between these signaling pathways in pathogenic fungi. An increasing body of evidence suggests the existence of crosstalk between autophagy and endocytosis, and some genes have been identified to be involved in both autophagy and other pathways (Table 3). For example, Atg6/Vps30/Beclin1, a key regulator of autophagy, is a subunit of different phosphatidylinositol 3-kinase complexes involved in either autophagy or vacuolar protein sorting via endosomes [56]. UVRAG, Vps38 human orthologues and Beclin1-binding proteins play roles in endocytosis and autophagy [57]. In yeast, the Atg6/Vps30 protein can form two different phosphatidylinositol-3-kinase (PI3K) complexes, type I and type II, which function in autophagy and vacuolar protein sorting, respectively [58]. Similar to yeast, MoAtg14 interacts with MoAtg6, which indicates that the type I PI3-K complex is conserved in *M. oryzae* [35]. MoVps38 demonstrates high similarity to UVRAG and interacts with MoAtg6 via a coiled-coil domain. PoVps9 recruits PoVps34 and targets its transport to endosomes by activating PoVps21 (also called PoRab5 in *M. oryzae*). MoAtg6 is then recruited by PoVps34 under the action of PoVps38 to induce endosomal endocytosis. Simultaneously, MoAtg6 is recruited to the PAS by PoVps34 to participate in the autophagy pathway by activating PoVps21 [19]. In addition, some Rab proteins, such as PoRab5 and MoYpt7, which function in the fusion of either late endosomes or autophagosomes with vacuoles, are involved in the regulation of autophagy in *M. oryzae* (Figure 4(a)) [37]. The Rab11A-positive compartment is a primary platform for autophagosome assembly mediated by the recognition of PI3P-RAB11A by WIPI2 [41]. In addition, some ATG proteins are involved in the apoptosis pathway in mammals: the conjugation of Atg12 with Atg3 inhibits mitochondrial fission and apoptosis, independent of autophagy, and Bcl-2 interacts with Beclin-1 to inhibit autophagy [59]. However, none of the available lines of evidence directly reveals the interrelations between autophagy and apoptosis in filamentous fungi. Other signaling pathways that regulate autophagy include the phosphoinositide-3-kinase (PI3K)/Akt/mTOR pathway, the Ras pathway, the MAPK and the Pmk1 MAPK pathway (Figure 4(b)) [2,14,60,61].

**Table 3.** Proteins with dual roles in autophagy and endocytosis.

Protein	Species	Functions	References
PoVps21	*Magnaporthe oryzae*	Involved in autophagic closure and the formation of the early endosome	[19,54]
MoYpt7	*Magnaporthe oryzae*	Essential for autophagic fusion and required for late endosome formation	[37]
MoAtg6	*Magnaporthe oryzae*	Primary cellular activator of autophagy and endocytosis	[19]
MoVps35	*Magnaporthe oryzae*	Involved in the origin of the autophagosomal membrane and endosome formation	[42]
MoEnd3	*Magnaporthe oryzae*	Mediates endocytosis and autophagy	[]

## Conclusions and perspectives

Although the autophagy pathway is a conserved process in eukaryotes, some differences have been found between *M. oryzae* and yeast/mammals. First, multiple PAS-like sites are found in each cell of the *M. oryzae* conidia, whereas only one PAS exists in *S. cerevisiae* [45]. In addition, Atg1 and Atg13 are conserved from yeast to mammals and form the Atg1-Atg13 complex, which is essential for autophagy in the yeast system [62,63]. However, the *MoATG13* mutant displays phenotypes similar to that of the wild-type strain Guy11, and the expression profiles of EGFP-MoAtg9 and DsRed2-MoAtg8 in the *MoATG13* mutant are comparable to those of the *MoATG9*-deletion mutant. We hypothesize that MoAtg13 loses its function during the autophagy process of *M. oryzae* and that the Atg1-Atg13 complex might not have been preserved during evolution [45]. Second, the basal level of autophagy under normal conditions is stronger in *M. oryzae* than in yeast, but under induction conditions, yeast exhibit reduced autophagy. In addition, several proteins have double or multiple functions and are involved in crosstalk between autophagy and other pathways [17,19,53,,64]. These results have not been reported in *S. cerevisiae*. Although the autophagy process has been well studied, the following questions require additional research: What is the origin of the autophagic membrane? Where does the energy required for autophagic formation originate? How do autophagic bodies (autophagosomes) move within the cell? Can autophagosomes travel between cells?

A key challenge for autophagy in plant-pathogen interactions is the accurate regulation of autophagy by pathogenic fungi at different phases. This is of particular interest during pathogen invasion because the roles of the autophagy pathway in the expansion of the pathogen within the host remain unknown. The appressoria of *M. oryzae* are currently being investigated using metabolomics and proteomics approaches, and the identification of new pathways or novel metabolites will help clarify the process and functions of autophagy in plant-pathogen interactions.
